# Differential Incorporation of β-actin as A Component of RNA Polymerase II into Regulatory Regions of Stemness/Differentiation Genes in Retinoic Acid-Induced Differentiated Human Embryonic Carcinoma Cells 

**DOI:** 10.22074/cellj.2016.4316

**Published:** 2016-05-30

**Authors:** Khadijeh Falahzadeh, Maryam Shahhoseini, Parvaneh Afsharian

**Affiliations:** 1 Department of Genetics, Reproductive Biomedicine Research Center, Royan Institute for Reproductive Biomedicine, ACECR, Tehran, Iran; 2 Department of Cell and Molecular Biology, Faculty of Biological Sciences, Kharazmi University (TMU), Tehran, Iran

**Keywords:** Nuclear Actin, NTera2/NT2, Retinoic Acid, Differentiation

## Abstract

**Objective:**

Nuclear actin is involved in transcription regulation by recruitment of histone modifiers and chromatin remodelers to the regulatory regions of active genes. In recent years, further attention has been focused on the role of actin as a nuclear protein in transcriptional processes. In the current study, the epigenetic role of nuclear actin on transcription regulation of two stemness (*OCT4* and *NANOG*) and two differentiation) *NESTIN* and *PAX6*) marker genes was evaluated in a human embryonal carcinoma cell line (NT2) before and after differentiation induction.

**Materials and Methods:**

In this experimental study, differentiation of embryonal cells was induced by retinoic acid (RA), and quantitative real-time polymerase chain reaction (PCR) was used to evaluate differential expression of marker genes before and 3 days after RA- induced differentiation. Chromatin immunoprecipitation (ChIP) coupled with real-time PCR was then undertaken to monitor the incorporation of β-actin, as a functional component of RNA polymerase II, in the regulatory regions of marker genes.

**Results:**

Data showed significant change in nuclear actin incorporation into the promoter regions of *NESTIN* and *PAX6* after RA-induction.

**Conclusion:**

We emphasize the dynamic functional role of nuclear actin in differentiation of embryonal cells and its role as a subunit of RNA polymerase II.

## Introduction

Actin, a ubiquitous protein of eukaryotic cells, is extremely conserved during evolution (1). So far, six actin isoforms have been identified in vertebrates with four muscle types (skeletal, cardiac, aorta-type smooth muscle, and stomach-type smooth muscle actins), and two non-muscle isoforms socalled cytoplasmic isoforms (cytoplasmic βand γ-actins) (2). Actin was traditionally considered as a cytoplasmic protein since it is involved in a wide range of cytoplasmic-related processes including cell motility, contractility, mitosis and cytokinesis, intracellular transport, endocytosis and secretion (3). However, based on the emerging evidences on the presence of actin in nuclear complexes, several fundamental roles of actin have become clear in nuclear processes. To date, it has been shown that actin is engaged in key nuclear processes such as transcription, mRNA processing and chromatin remodeling (4). 

Several reports have shown the involvement of actin in transcription by all three RNA polymerases I, II and III (5,8). Based on the association of actin-like MreB protein with E.coli RNA polymerase, it has been suggested that interaction of actin with RNA polymerase could be conserved from prokaryotic to eukaryotic organisms (8). Interestingly, Kukalev et al. (9) clearly demonstrated the association of actin with the carboxy terminal domain (CTD) of the largest subunit of RNA polymerase II. With respect to the absence of this subunit in other polymerases, this interaction made a particular actin binding site to RNA polymerase II. 

Presence of actin in preinitiation complexes (PIC) and also *in vitro* experiments of blocked transcription initiation in the absence of β-actin suggest that actin is crucial for the assembly of transcription-competent polymerases (5,6,10,11). Given that the control of transcriptional activities has a key role in differentiation and developmental process, presence of β-actin in transcription-competent polymerases may be another level of regulating cell differentiation. 

Based on immunoreactivity and mass spectrometry criteria, it was shown that among the six actin isoforms, only β-actin is a component of RNA Polymerase II, heterogeneous nuclear ribonucleoproteins (hnRNPs) and proteins associated with nascent transcripts (6,7,12). Working on HeLa cells, chromatin immunoprecipitation (ChIP) assays have demonstrated the presence of actin at the promoter regions of several inducible genes in this cellular system, hence the relevance of actin with transcription (6,13). It is therefore suggested that actin or actin like proteins have functional roles in the transcriptional machinery of living cells. 

To better understand the potential role of β-actin in the differentiation process, comparative incorporation of β-actin into promoters of inducible marker genes, with different expression profiles in pluripotency and differentiation, was considered worthwhile to investigate. Accordingly, a human embryonic carcinoma cell line, namely NTera2/ NT2, was used as an embryonal model system which can undergo differentiation under retinoic acid (RA) induction. Embryonic carcinoma cell lines derived from germ cell tumors are valuable models for elucidating molecular mechanisms involved in differentiation and developmental biology processes (14,15). 

In the current study, the epigenetic role of nuclear actin was assessed on transcriptional regulation of *OCT4* and *NANOG* as two stemness marker genes, and *NESTIN* and *PAX6* as two differentiation marker genes before RA induction and 3 days after. Although integration of β-actin in the promoter region of several inducible genes has been shown previously (6), to the best of our knowledge, this study is the first in which differentiationsensitive alterations in β-actin incorporation has been checked. 

## Materials and Methods

### Cell culture

NTera2 clone D1 (NT2.cl.D1, a gift from Dr. Peter Andrews) embryonal carcinoma (EC) cells were grown in Dulbecco’s modified Eagle’s medium (DMEM), containing 10% fetal calf serum (FCS) and 1% penicillin/streptomycin mixture (16). The cells were maintained in 5% CO_2_ atmosphere at 37˚C and were treated with 10 µM of RA to trigger the differentiation process. A monolayer of pluripotent cells were harvested as the cellular source for untreated cells (day 0) and RA-induced cells were harvested on day 3 of differentiation. Both cell groups were stored at -80˚C for molecular analyses. 

### RNA isolation and quantitative real-time polymerase chain reaction

Total RNA isolation and cDNA synthesis were performed on harvested cells as previously described (16,17). Synthesized cDNA from 2 μg of total RNA was amplified with specific sense/antisense primers given in Table 1. 

Gel electrophoresis was carried out on a 1.7% agarose gel stained with ethidium bromide (10 µg/ ml) and polymerase chain reaction (PCR) products visualized by UV transluminator ( Molecular Imager® Gel Doc™ XR+ (BioRad, USA). Real-time PCR was performed on an ABI 7500 real-time PCR using SYBR green mastermix and standard ABI cycling conditions. Differential expression was analyzed using the 2^-ΔΔct^ quantitative method (18) to estimate relative fold-change in expression. *GAPDH* expression level was considered as the reference gene for expression normalization. 

**Table 1:** Primer pairs used in this study


Gene	Primers (5´→3´)	Annealing temperature (˚C)	Product size (bp)

RT-PCR and real-time PCR primers			
*GAPDH*	F: CTCATTTCCTGGTATGACAACGA	60	122
	R: CTTCCTCTTGTGCTCTTGCT		
OCT4	F:GTTCTTCATTCACTAAGGAAGG	60	101
	R: CAAGAGCATCATTGAACTTCAC		
NANOG	F:AAAGAATCTTCACCTATGCC	60	110
	R: GAAGGAAGAGGAGAGACAGT		
NESTIN	F:TCCAGGAACGGAAAATCAAG	60	120
	R: GCCTCCTCATCCCCTACTTC		
PAX6	F:GTCCATCTTTGCTTGGGAAA	60	110
	R: TAGCCAGGTTGCGAAGAACT		
ChIP-real-time PCR primers			
OCT4	F: GAACTTCTAACCTTCATAACCTG	60	157
	R: CATTCACCCATTCCCTGTTC		
NANOG	F:AATTCACAAGGGTGGGTCAG	60	133
	R: TAACATGAGGCAACCAGCTC		
NESTIN	F:GCTCGCAGAGCTTTTAGGAC	60	188
	R: GCTGCCACTCTCTGACCTCT		
PAX6	F:CCTGCCCCAGAGTTTAAATG	60	81
	R: GCTGGCGTGGATATTAAGGA		


RT-PCR; Reverse transcriptionpolymerase chain reaction and ChIP; Chromatin immunoprecipitation.

### Chromatin immunoprecipitation coupled with real-time polymerase chain reaction

ChIP experiments were carried out using the Orange ChIP kit (Diagenode, Belgium) as described before (15). Cross-linked chromatin obtained from 1×10^5^ harvested cells was immunoprecipitated with anti-β-actin (Sigma cat # A1978, USA) and anti-RNA polymerase II (AbcamR cat # Ab5408, UK) antibodies. The precipitated DNA was analyzed by real-time PCR using specific primers for promoters of marker genes as listed in Table 1. Data were expressed as fold enrichment of DNA associated with immunoprecipitated enriched-DNA relative to a 1/100 dilution of input chromatin.

### Statistical analysis

All data were analyzed using repeated-measures followed by Least Significant Difference (LSD) test for pairwise comparisons to day 0. The significance level was set at P<0.05. Bars and Error bars represent mean ± SD. The Statistical Package for Social Sciences (SPSS) version 16 program was used for all statistical analysis.

## Results

### Differential expression of marker genes in NT2 cells under retinoic acid-induced differentiation

**Fig.1:**
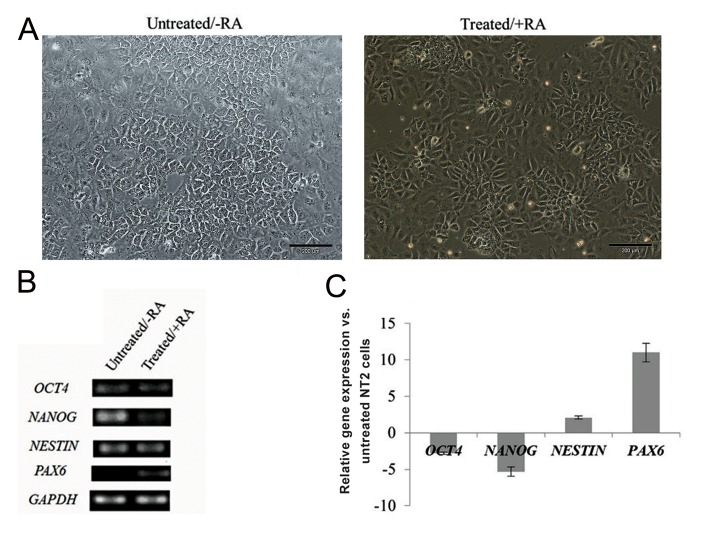
Differentiation induction of human embryonal carcinoma cells (NT2) by retinoic acid (RA). A. Phase contrast microscope images of NT2 cells in the untreated state and after 3 days of RA induction, B. Expression detection of *OCT4* and *NANOG* transcripts as pluripotency marker genes and *NESTIN* and *PAX6* transcripts as differentiation marker genes during NT2 cell differentiation (RA represents NT2 cells without RA treatment and +RA represents NT2 cells after RA induction). Expression of the housekeeping gene *GAPDH* was used as an internal control and C. RA-induced changes in quantitative gene expression of the marker genes throughout differentiation of NT2 cells. NT2 cells were treated with RA and gene expression of samples at different stages was relatively quantified by real-time polymerase chain reaction (PCR) analysis before and 3 days after differentiation. The results are expressed as the log of normalized relative fold changes in activity (mean ± SD).

Using a previously established protocol, we induced NT2 cells toward differentiation (Fig.1A). Our results from reverse transcription quantitative PCR revealed that transcript levels of the two stemness marker genes, OCT4 and NANOG, were down-regulated after 3 days of differentiation. Moreover, *NESTIN* showed a low expression level in undifferentiated NT2 cells and was up-regulated in treated cells. Consistently, no expression of *PAX6* was detected in undifferentiated cells, however, a remarkable increase was observed for this developmental marker gene after RA induction (Fig.1B,C). The expression pattern therefore confirmed trigger of differentiation in NT2 cells under RA treatment. 

### Decreased incorporation of β-actin into regulatory regions of differentiation marker genes

**Fig.2:**
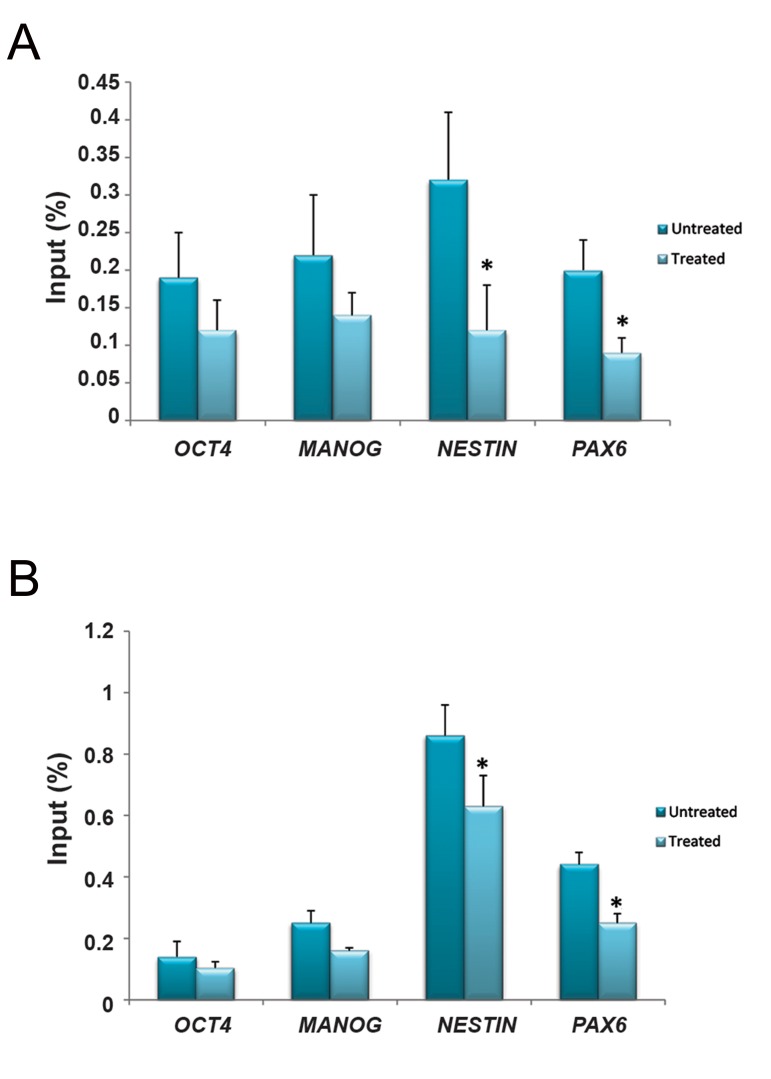
Chromatin immunoprecipitation analysis of incorporation of A. β-actin and B. RNA polymerase II into promoter regions of the marker gene set in NT2 cells in untreated state and after 3 days of RA induction. The results are represented as fold enrichment of DNA associated with indicated immunoprecipitated proteins to a 1/100 dilution of input chromatin (chromatin without antibody) (mean ± SD, *; P<0.05).

Although nuclear actin is basically considered as a functional component of RNA polymerase II, chromatin immunoprecipitation analyses were performed with respect to cell differentiation. The presence of β-actin and RNA polymerase II was monitored in regulatory regions of the four marker genes before and after RA treatment. As shown in Figure 2, incorporation of both β-actin and RNA polymerase II decreased through induction of differentiation but were significant for *NESTIN* and *PAX6* promoters (P<0.05). 

## Discussion

In this study, the epigenetic role of β-actin was studied in NT2 embryonal carcinoma cells by specifically examining its incorporation into promoter regions of four embryonic/developmental marker genes. Given that EC cells are considered as pluripotent cell lines, activation of pluripotency marker genes such as *OCT4* and *NANOG* are required for maintenance of their pluripotency and self-renewal (19). On the other hand, induction of differentiation triggers down-regulation of these stemness marker genes in parallel to up-regulation of the developmental genes which are normally suppressed in undifferentiated cells (16,17,20). 

Several reports have demonstrated that actin is associated with all three RNA polymerases, (5,7,9,21) and co-immunoprecipitation experiments have also shown that actin can interact with the CTD of the largest RNA Polymerase II subunit (9,21). With respect to a number of studies which revealed the presence of actin in promoters of RNA Polymerase II-transcribed genes and its co-purification with PIC, it is suggested that actin is required for the assembly of transcription machinery at promoters (5,6,10,11). In this study, ChIP-real-time primers were designed for promoter regions of all four marker genes and due to the fact that formation of preinitiation complex occurrs at promoters, we detected incorporation of β-actin in parallel with incorporation of RNA polymerase II into these regulatory regions. Our results were consistent with the role of β-actin as a component of RNA polymerase II because of its presence at promoters of genes transcribed by RNA Polymerase II. 

After RA induction of EC cells, pluripotency and differentiation marker genes were downand up-regulated respectively. Accordingly, we expected higher incorporation of RNA polymerase II and β-actin into differentiation gene promoters and less into pluripotency genes after RA induction. 

Contrary to our expectation, a significantly lower level of RNA polymerase II and β-actin incorporation was observed into regulatory regions of *NESTIN* and *PAX6*. This may point out that the target genes are not regulated at the level of PIC assembly. 

Until two decades ago, it was generally accepted that formation of PIC resulted in the recruitment of RNA Polymerase II to promoters and was considered as a main step of transcriptional regulation (22). Some recent reports, however, have shown that post initiation regulation is more common than the old notion about the fundamental role of PIC assembly in transcriptional regulation (23). These reports proposed that not only transcriptionally active genes but also silent genes can display signs of transcription initiation (as shown here for the silent genes *OCT4* and *NANOG*), and formation of preinitiation complex is not the rate-limiting step in regulating transcription of many genes. 

Studies have illustrated that transcriptionally inactive promoters (poised promoters) are found across a wide range of organisms from bacteria to humans (24,26). Whole-genome studies have demonstrated that a majority of developmental and stress-inducible genes were preloaded with RNA polymerase II at promoter-proximal regions (27,28) and a ChIP-chip study also showed that a large number of genes in human embryonic stem cells contain poised promoters loaded with polymerases (27). The frequent poised polymerases on developmental genes strongly suggest that the key rate-limiting step of the regulation of development could be transcription elongation instead of the PIC formation step (29). 

Although our data showed undetectable expression of *PAX6* in untreated NT2 cells, its promoter was occupied with RNA polymerase II and β-actin according to the ChIP-real-time PCR assay. Furthermore, since the embryonal carcinoma cell line has very similar properties to embryonic stem cells, the presence of RNA polymerase II and β-actin on the promoters of all four marker genes is merely a preloaded structure regardless of the activity of the genes. Therefore, when transcription significantly increases in the interval between preand post-induction, occupancy of a large portion of the transcription machinery has already happened (30). Based on declined amounts of RNA polymerase II and β-actin observed on *NESTIN* and *PAX6* promoters of post-induction cells, it can be suggested that entrance of transcriptional machineries toward the elongation phase as a consequence of upregulation of these genes probably leads to reduced occupancy of these promoters by RNA polymerase II and β-actin. 

## Conclusion

Our data support the functional role of β-actin in transcription as a component of RNA polymerase II and its dynamicity in pluripotency/differentiation switch of NT2 embryonal carcinoma cells. This study emphasizes the importance of the newly accepted notion of post-initiation regulation of transcription during development of embryonal cells. 
